# Neural Connectivity Underlying Reward and Emotion-Related Processing: Evidence From a Large-Scale Network Analysis

**DOI:** 10.3389/fnsys.2022.833625

**Published:** 2022-04-07

**Authors:** Ala Yankouskaya, Toby Denholm-Smith, Dewei Yi, Andrew James Greenshaw, Bo Cao, Jie Sui

**Affiliations:** ^1^Department of Psychology, Bournemouth University, Bournemouth, United Kingdom; ^2^School of Natural and Computing Sciences, University of Aberdeen, Aberdeen, United Kingdom; ^3^Department of Psychiatry, Faculty of Medicine & Dentistry, Edmonton, AB, Canada; ^4^School of Psychology, University of Aberdeen, Aberdeen, United Kingdom

**Keywords:** self-prioritization, reward processing, emotion processing, default mode network, frontoparietal network, salience network, interaction

## Abstract

Neuroimaging techniques have advanced our knowledge about neurobiological mechanisms of reward and emotion processing. It remains unclear whether reward and emotion-related processing share the same neural connection topology and how intrinsic brain functional connectivity organization changes to support emotion- and reward-related prioritized effects in decision-making. The present study addressed these challenges using a large-scale neural network analysis approach. We applied this approach to two independent functional magnetic resonance imaging datasets, where participants performed a reward value or emotion associative matching task with tight control over experimental conditions. The results revealed that interaction between the Default Mode Network, Frontoparietal, Dorsal Attention, and Salience networks engaged distinct topological structures to support the effects of reward, positive and negative emotion processing. Detailed insights into the properties of these connections are important for understanding in detail how the brain responds in the presence of emotion and reward related stimuli. We discuss the linking of reward- and emotion-related processing to emotional regulation, an important aspect of regulation of human behavior in relation to mental health.

## Introduction

Neurobiological mechanisms underlying motivational factors (including reward, emotion, self-relevance) that influence goal-oriented cognition and behavior are of major interest in translational clinical and cognitive research. Well-documented studies indicate that dysfunction in emotion- and reward-related processing may be a prominent transdiagnostic feature for a number of psychiatric disorders (Ryan and Skandali, 2016; Sabharwal et al., 2016; Zhang et al., 2016; Barkus and Badcock, 2019; Scalabrini et al., 2020). Recent theoretical and empirical work has advanced our understanding of the neural underpinnings of emotion and value-based reward processing, including findings on social motivation and associated brain functions (Palminteri et al., 2015; Kragel and LaBar, 2016; Young et al., 2016; Fox, 2018; Hoemann et al., 2020; Jauhar et al., 2021). However, important questions remain concerning the intersection between reward and emotion at the neural level (Sander and Nummenmaa, 2021).

The effects of value-based reward- and emotion-related processing share many commonalities in their influence on cognition and goal-directed behavior. For example, both the presence of reward- and emotion-related stimuli generate robust facilitation effects on visual attention selection (Anderson and Yantis, 2013; Ono and Taniguchi, 2017; Stolte et al., 2017, 2021); enhance perceptual learning (Fox et al., 2002; Anderson et al., 2011; Sui et al., 2015b; Watson et al., 2020) and carryover effects (Fiori and Shuman, 2017; Vartak et al., 2017). These commonalities between them have been conceptualized in several theoretical accounts of emotion as an emergent property of motivationally driven neural activity (Panksepp, 1998; Buck, 2000; Laming, 2000; Lang and Bradley, 2008; Pessoa, 2009). Support for such accounts came from multiple meta-analyses indicating that neural processes triggered by motivational stimuli may overlap in the cingulate cortex, anterior insula, ventral striatum, dorsolateral, and ventromedial prefrontal cortices (Bartra et al., 2013; Lindquist et al., 2016; Cromwell et al., 2020). Engagement of these regions was observed across a range of reward and emotion tasks regardless of emotional valence (i.e., positive or negative) of stimuli, leading to a hypothesis that the same underlying system is responsive to the basic properties of general affect (Lindquist et al., 2016; Park et al., 2019).

Although attractive, testing this hypothesis yielded inconsistent empirical results. Activation studies that aimed to directly examine common and distinct neural processes triggered by emotional and value-based reward stimuli reported no evidence for between-task overlap, indicating dissociable neural processes for reward and emotion (Park et al., 2018). Assessment of whether positive and negative reward-predictive emotional stimuli would lead to compatible effects based on the integration of overlapping and non-overlapping basic valence did not produce a conclusive result. For example, positive reward vs. positive no-reward stimuli failed to yield differential neural activation, whereas some areas in the inferior frontal gyri, superior medial gyrus, left insula, and the inferior parietal lobule responded to interaction between reward and negative emotion (Park et al., 2019). Combined meta-analytical and empirical procedures have addressed common neural mechanisms of reward and emotion processing by investigating their influence on cognitive control (Brandl et al., 2019). Indirectly supporting the initial hypothesis, a large overlap of cognitive reward- and emotion-control activation patterns has been reported, suggesting a common mechanism for the control of motivational and emotional states (Brandl et al., 2019). Specifically, a significant overlap was found across sensomotor areas, dorsolateral prefrontal cortex (dLPFC), ventrolateral prefrontal cortex (vLPFC), dorsomedial prefrontal cortex (dMPFC), anterior insula, and parietal cortices. This common reward/emotion activation pattern may be defined by intrinsic co-activity networks mapped into domain-general networks, such as frontoparietal (FPN), default-mode (DMN), cingulo-opercular (or Salience, SN), and dorsal attention networks (DAN; Brandl et al., 2019), that drive motivated behavior (Sui and Gu, 2017).

The involvement of these FPN, DMN, SN, and DAN has been conceptualized in the Triple Network Model of psychopathology (Menon, 2011). The model proposed that deficits in engagement and disengagement of these three core networks plays a significant role in many psychiatric and neurological disorders with symptomatic deficits in reward- and emotion-related processing (e.g., schizophrenia, bipolar disorder, major depression). Several recent studies reported involvement of these intrinsic brain networks in value-based reward or emotion processing in healthy individuals (Jiang et al., 2018; Pan et al., 2018; Lin et al., 2019; Flannery et al., 2020; Grill et al., 2021) and aberrant functioning of these networks in patients (Whitton et al., 2015; Alegria et al., 2016), demonstrating a general agreement on the importance of the DMN, DAN, SN, and FPN in processing reward- and emotion-related information. These studies, however, fall short of providing an understanding of how these large-scale neural networks interact to support value-based reward or emotion processing. Questions remain unanswered concerning whether the brain forms the same components of interconnected networks for prioritizing value-based reward and emotional information. There is also a lack of understanding on how networks associated with value-based reward overlap with those associated with emotion; whether value-based reward engages the same networks as positive emotion processing or are distinct separate networks involved in reward- and emotion-related processing.

Analysis of large-scale neural networks has proven useful for a better understanding cognitive functioning in healthy populations and patients (Bassett and Sporns, 2017; Shi et al., 2021; Zhu et al., 2021). The underlying assumption of this approach is that the brain’s functional network architecture during task performance is shaped primarily by intrinsic networks which are temporally correlated during different tasks (Smith et al., 2009; Cole et al., 2014, 2016). The large-scale neural network analysis approach can be used to identify the structure of interconnected intrinsic networks (also knowns as topological clusters) among the set of all connections between brain networks. We applied this approach to fMRI data where healthy young adults performed value-based reward- and emotion-associative matching tasks which have previously been shown to yield reliable prioritized responses to emotion- and reward-related stimuli (Sui et al., 2012; Yankouskaya et al., 2017). Overcoming methodological issues in previous research, experimental procedures in our tasks followed an identical procedure differing only in stimuli to elicit value-based (high reward value vs. low reward value) or emotion (happy vs. neutral, sad vs. neutral) effects. We mapped these effects separately onto a set of brain networks to identify topological clusters among the set of all connections using a network-based statistics approach (NBS; Zalesky et al., 2010). To resolve inconsistent findings regarding the role of brain networks in reward and emotion-related processing, we tested two hypotheses using this large-scale neural network analysis approach. Based on the previous research (Lindquist et al., 2016; Park et al., 2019), our primary hypothesis is that value-based reward engages the same set of interconnected networks as positive emotion processing. Accepting this hypothesis would provide further evidence for the overlap between brain systems responsive to the basic properties of general affect. Alternatively, the brain may form partly overlapping but different components of interconnected networks such as DMN, DAN, SN, and FPN to prioritize value-based reward and emotional information. Accepting the alternative hypothesis would inform the Triple Network Model of psychopathology (Menon, 2011) by identifying components sensitive to context-specific motivational information.

## Materials and Methods

### Datasets and Experimental Design

**Figure 1:**
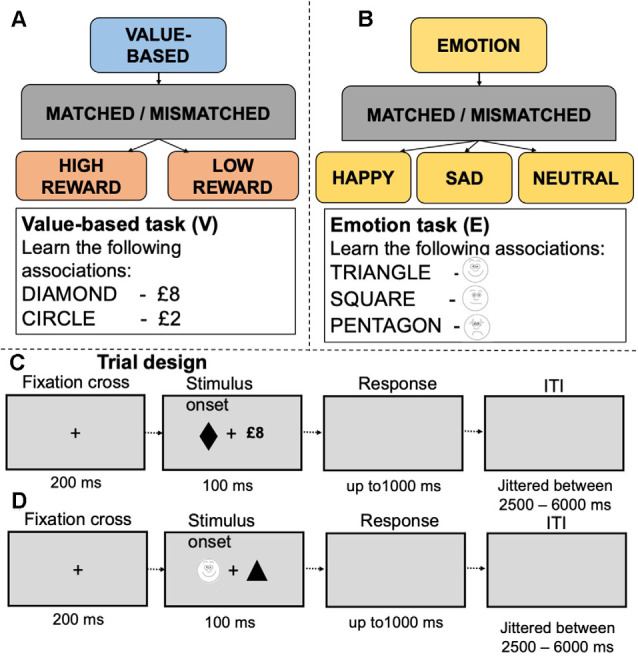
Experimental design and examples of stimuli in the value-based **(A)** and emotion **(B)** tasks. Panels **(C,D)** depict experimental trials for the value-based and emotion tasks respectively.

We employed fMRI data from two experiments where young adults performed emotion- (Yankouskaya and Sui, 2021), dataset 1 (21 participants, 10 males, 11 females, age *M* = 23.6, SD = 2.8) and value-based reward (Yankouskaya et al., 2017, dataset 2; 19 participants, nine males, 10 females, age *M* = 25.8, SD = 7.31) associative matching tasks. The idea of this task is to associatively “tag” a basic stimulus such as a simple geometrical shape with value-based reward or emotion information. Following this “tagging,” perceptual responses to a stimulus associated with higher reward value or emotionally valenced information enhanced (Sui et al., 2012; Stolte et al., 2017). The procedure offers tight control over confounding factors (such as complexity and familiarity of stimuli). In the emotion task, 21 individuals learned associations between simple geometrical shapes and schematic emotional expressions depicting sadness, happiness and neutral emotional expression. After the learning stage (1–2 min), participants performed a matching task indicating whether a displayed pairing matched or mismatched the learned associations. In the value-based task, nineteen participants learned associations between simple geometrical shapes (e.g., square, circle) and value labels (e.g., square—£8, circle—£2). After the learning stage, they performed “shape-label” matching, indicating whether a presented shape-label pair matched or mismatched associations learned earlier (Figure 1).

Both the emotion and value-based tasks followed an identical experimental protocol. Geometric shapes (circle, hexagon, square, rectangle, diamond and triangle) were randomly assigned to conditions in each task. The stimulus display contained a fixation cross (0.8°×0.8°) at the center of the screen with a shape (3.8°×3.8°) and a label on either side of fixation. The distance between shape and label was 10°. Presentations of the shapes and labels were counterbalanced across trials. Each trial started with a fixation cross for 200 ms, followed by the stimulus display for 100 ms and a blank interval which remained for 1,000 ms. Trials were separated by a jittered interstimulus interval (ranging between 2,000 and 6,000 ms). In each study, before entering the scanner, participants performed a short practice session (12 trials per task). Feedback on accuracy (words “Correct!” or “Incorrect!”) and overall response time were provided after each trial during practice only (detailed information about these tasks is presented in Supplementary Table 1). Participants’ responses were recorded using an MRI compatible response box and controlled by Presentation software^1^. In both studies, participants reported no use of psychotropic medications or past diagnoses for psychiatric, neurological disorders and have normal or corrected-to-normal vision size. As a part of the pre-screening procedure of dataset 1, participants completed the Mood and Anxiety Symptom Questionnaire (MASQ), a 77-item self-report questionnaire that assesses depressive, anxious and mixed symptomatology. Only participants with low scores on each of five subscales were invited to the scanning session. Participants in dataset 2 received a monetary incentive for correct responses to matched trials. The monetary incentives were scaled according to the value assigned to a shape (2%) and implemented to ensure reward motivation. The monetary incentives were not presented on the screen during the task and were paid off after the experiment was completed as a lump sum.

Imaging data acquisition for each dataset is summarized in Supplementary Table 2. Both studies were approved by the Central University of Oxford Research Ethics Committee (CUREC). All participants provided written informed consent.

### Behavioral Data Analysis

In each task, we measured accuracy (percent correct responses) and response times. Here we report data analysis for matched trials only (full data analyses are presented in Supplementary Table 3). Only correct responses were used for reaction time analyses. A one-way repeated measures ANOVA was carried out to examine the effect of stimuli on response time in each task.

### fMRI Data Preprocessing

The datasets were pre-processed and analyzed separately using SPM12 (Wellcome Trust Centre for Neuroimaging, London, UK^2^) running in Matlab R2021a (Mathworks, Inc., Natick, MA, USA). The pre-processing pipeline, modeling and analytical steps were identical for each dataset. Pre-processing included slice-timing correction, functional realignment and unwarp, segmentation and normalization. First, all scans were corrected for differences in slice acquisition times to make the data on each slice correspond to the same point in time. Slice timing correction was performed using the middle slice as reference. Next, the data were aligned across and within functional sessions and unwarped using a least squares approach and a 6-parameter spatial transformation. Anatomical data were registered to the first functional frame and spatially normalized to Montreal Neurological Institute (MNI) space using SPM12 unified segmentation–normalization algorithm (Ashburner and Friston, 2005). Functional data were resampled to a 91 × 109 × 91 bounding box with 2 mm isotropic voxels. No additional spatial smoothing was applied to minimize artificial local spatial correlations in the whole-brain analysis.

After the initial pre-processing in SPM12, the datasets were submitted separately to the CONN toolbox (version 20a) for additional denoising steps and functional connectivity analyses. First, we used the ART procedure implemented in CONN for artifact detection. The results of gross head movements detection indicated that our sample did not contain participants with a head displacement exceeding 3 mm in more than 5% of volumes in any sessions. It has been suggested that functional connectivity can also be influenced by small volume-to-volume “micro” head movements (Van Dijk et al., 2012). To ensure that micro-head movement artifacts did not contaminate our findings, functional data with frame-to-frame displacements greater than 0.40 mm were censored (Power et al., 2014). After the denoising step, we performed a quality control (QC)–functional connectivity (FC) check implemented in CONN to assess residual effects of subject motion (Ciric et al., 2017). This method computes functional connectivity between randomly selected pairs of points within the brain and evaluates whether these connectivity values are correlated with other QC measures such as subject-motion parameters. The QC-FC showed that the QC-FC 100% matched with the null hypothesis indicating that functional connectivity did not associate with the residual effects in both datasets (Supplementary Figure 1).

Recent studies showed that FC results can be severely affected by physiological noise (Birn et al., 2014). To address this issue, we used an anatomical Component based noise Correction method (aCompCor, Behzadi et al., 2007) that derives potential physiological and movement effects on the BOLD timeseries by evaluating the signal within white matter and CSF areas. It was suggested that this method does not suffer severely from systematic introduction of negative correlation (Murphy et al., 2009) while retaining some of the advantages of global signal regression (GSR; Chai et al., 2012). The principal components of the signal from eroded white matter and CSF masks were regressed out. However, GSR was not performed due to the ongoing controversy associated with this step (Caballero-Gaudes and Reynolds, 2017) and recent evidence that removing global signal eliminates an important source of neural activity when assessing FC between networks (Scalabrini et al., 2020). The noise components from white matter and CSF, estimated subject-motion parameters (three rotation, three translation parameters plus their associated first-order derivatives) and outlier scans were regressed out as potential confounding effects. We also included session and task effects as additional noise components to reduce the influence of slow trends and constant task-induced responses in the BOLD signal (Cole et al., 2019). Finally, a high-pass filter (e.g., *[0.008 inf]* which implements the standard 128 s high-pass used in SPM for regular task analyses) was applied to functional data as an acceptable compromise between minimizing cross-talk/spillage of the BOLD signal between session/conditions while still benefiting from the increased SNR afforded by filtering.

### Network Analysis

After the pre-processing and denoising steps, the residual time series from each run within each dataset were concatenated to form a condition-specific time series of interest. For the first-level analysis, we used ROI-to-ROI connectivity (RRC) measures of large-scale networks. The large-scale networks ROIs were defined from default CONN’s networks atlas (derived from ICA analyses based on the Human Connectome Project (HCP) dataset of 497 subjects). The networks atlas delineates an extended set of 32 classical networks: Default Mode Network (four ROIs), SensoriMotor (two ROIs), Visual (four ROIs), Salience/Cingulo-Opercular (seven ROIs), DorsalAttention (four ROIs), FrontoParietal/Central Executive (four ROIs), Language (four ROIs), Cerebellar (two ROIs). The Cerebellar ROIs (Anterior and Posterior) were not included as it only had partial coverage in the participants. Detailed list of these network and their notes is provided in Supplementary Figure 2. In total, we analyzed 830 connections across 30 ROIs. However, rather than focusing on any of these networks in isolation, we treated all ROIs as “nodes” within a whole-brain network.

To define network components associated with value-based, emotion and valence processing, we used the network-based statistical analysis (NBS; Zalesky et al., 2010). First, we defined condition-specific functional connectivity strength (i.e., functional connectivity during each task/condition), by computing weighted RRC matrices using a weighted Least Squares linear model with temporal weights identifying individual experimental conditions of interest in each dataset. The weights were defined as a condition-specific boxcar timeseries convolved with a canonical hemodynamic response function. Weighted RRC matrices of Fisher-transformed bivariate correlation coefficients between all ROIs/nodes (30 × 30) were calculated for each task/condition/participant. These matrices were submitted to the second-level analysis where the differences between conditions constituting value-based-prioritization (high reward value > low reward value), emotion-prioritization (happy + sad > neutral) and valence-prioritization (happy > neutral, sad > neutral) were calculated for every edge/connection using a General Linear Model (GLM).

The resulting statistical parametric map for each contrast was thresholded using a priory connection threshold (“height” threshold; uncorrected *p* < 0.001) to construct a set of suprathreshold links among all ROIs/nodes of between-condition differences. It has to be noted that this connection threshold is a user-determined parameter in NBS analysis. It has been suggested that sensitivity to the connection threshold might reveal useful information about the nature of the effect (Zalesky et al., 2010). For example, effects presented at only conservative connection threshold (e.g., *p* < 0.001) are likely to be characterized by strong, topologically focal differences between conditions constituting the effect. Effects presented only at relatively liberal threshold (e.g., *p* < 0.05) are likely to be subtle yet topologically extended. Effects presented at both thresholds combine features of topologically focal and distributed differences. Although our analysis focused on the former threshold, we also explored changes in connectivity using the lower threshold. This evidence may be tested in future studies with large sample size.

Next, in the set of suprathreshold links, we identified any connected components (topological clusters) and defined the size of each component as the sum of T-squared statistics overt all connections within each component. The critical assumption inherent to the NBS here is that connections for which the null hypothesis is false are arranged in an interconnected configuration, rather than being confined to a single connection or distributed over several connections that are in isolation of each other. In other words, the presence of a component may be evidence of a non-chance structure for which the null hypothesis can be rejected at the level of the structure as a whole, but not for any individual connection alone (Fornito et al., 2015). Finally, a FWE-corrected p-value for each component were computed using permutation testing. The basic assumption of the permutation procedure is that under the null hypothesis, random rearranging correspondence between data points and their labels does not affect the test statistics. This would not be the case if the null hypothesis were false. The labels for each tested contrast (e.g., high reward value > low reward value) were randomly rearranged for corresponding data points according to a permutation vector of integers from 1 to the total number of data points. The same permutation vector was used for every connection (830 in total) to preserve any interdependencies between connections and constrained to remain within the same participant. The size of the largest component was recorded for each permutation yielding an empirical null distribution for the size of the largest component size. This procedure was performed 1,000 times. The FWE-corrected p-value for a component of given size was then estimated as the proportion of permutations for which the largest component was of the same size or greater and, thus, representing the likelihood under the null hypothesis of finding one of more components with this or larger mass across the entire set of networks.

To characterize the properties of each component, we report “size” as the number of suprathreshold connections, “intensity” (mass) measures as their overall strength (i.e., sum of absolute T-values over these suprathreshold connections) and p-values associated with these measures. In addition, we provide complementary statistics for each connection such as effect size for significant components calculated by averaging the test statistic values across significant connections and dividing by the square root of the number of subjects and between-subject variability for each connection within a component to gain more insight into contrasts of interest. It has to be noted that we do not violate the NBS inference about a component as a whole by providing effects sizes for each connection. The information about effect sizes helps to interpret the nature of connections within each component.

## Results

### Value-Base Reward and Emotion Prioritization Effects in Behavior

Participant’s responses in both tasks were accurate (95.23% in the reward task and 85.76% in the emotion task; for details see Supplementary Table 3).

**Figure 2:**
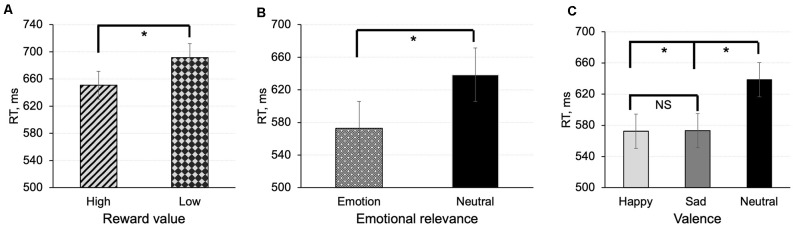
Mean reaction time in the value-based task **(A)** and emotion task **(B,C)** for matched (correct pairings) associations between shapes and labels. In the emotion task, we calculated effects of emotional relevance (averaged effect of emotions) **(B)**, and emotional valence **(C)**. The error bars represent ± SEM. *p < 0.05; NS, non-significant.

In the value-based task, there was a main effect of reward value [*F*_(1,18)_ = 6.61, *p* = 0.019, MD = −46.34, 95% CI (−58.98, −37.12)] indicating that participants were faster in responding to shapes associated with high reward value compared to shapes associated with low reward value (Figure 2A).

In the emotion task, there was a main effect of emotional relevance on response time [*F*_(1,20)_ = 62.64, *p* < 0.001, MD = −65.76, 95% CI (−87.45, −49.18)] indicating that participants responded faster to shapes associated containing emotional information than to neutral shapes (Figure 2B). Assessment of the effect of valence on response times revealed that reaction times for happy and sad associations were faster compared to associations with neutral emotional expression [*F*_(2,40)_ = 29.70, *p* < 0.001; *t*_(20)_ = −6.83, *p* < 0.001, MD = −69.38, 95%CI (−84.93, −47.35); *t*_(20)_ = −6.51, *p* < 0.001, MD = −66.14, 95% CI (−82.97; −47.78)]. The difference between happy and sad associations was not significant (*t*_(20)_ = −0.32, *p* = 0.75; Figure 2C).

### Functional Connections Explaining Value-Related Prioritization Effect

**Figure 3:**
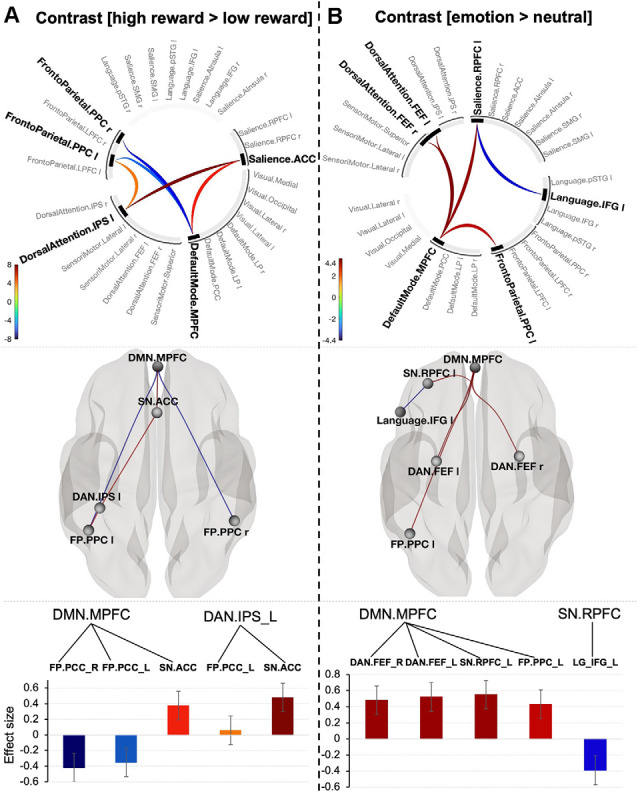
Connectogram of networks (top row), corresponding glass brain (middle row) and effect sizes of individual connections within a cluster (low row) for value-based **(A)** and emotion **(B)** prioritization effects [the connectivity threshold *p* < 0.001, cluster threshold p-FWE corrected (*p* < 0.05)]. Vertical color bars indicate T-test statistics for individual connections. Glass brain figures visualize spatial location of connections comprising each component where a sphere represents the center of the corresponding network. The red and blue lines depict positive or negative correlations between networks. Effect sizes: the Y axis represents Pearson correlation values where the sign indicates the direction of the effect. Error bars represent standard deviations. The color of the effect size bars corresponds to the color of relevant connections in the connectogram.

Contrast [high reward > low reward] for *p* < 0.001 connection threshold (uncorrected) and p-FEW corrected cluster threshold (*p* < 0.05) revealed one topological cluster (mass = 187.61, p-FWE = 0.001, size = 5; Figure 3A). In this cluster, the DMN (MPFC) showed positive connectivity with the Salience network (ACC; *t*_(18)_ = 5.82, p-FWE = 0.004) and negative connectivity with the Frontoparietal network bilaterally (posterior parietal cortex, PPC; *t*_(18)_ = −6.66, p-FEW = 0.001; *t*_(18)_ = −4.64, p-FWE = −4.64, p-FWE = 0.03 respectively for the right and left PPC). The left PPC of the Frontoparietal network and ACC part of the Salience network showed positive connectivity to DAN (left IPS), but the former connection was at the border of significance (*t*_(18)_ = 4.36, p-FWE = 0.046; *t*_(18)_ = 8.29, p-FWE = 0.0001 respectively).

No significant components were found when we decreased the connectivity threshold to *p* < 0.05. In contrast, systematically increasing the threshold by 10% showed that the effect occurred at only a conservative threshold (see details in Supplementary Table 4) indicating that the value-related prioritization effect is likely to be characterized by strong, topologically focal differences in functional connectivity.

### Functional Connections Explaining Emotion-Related Prioritization Effect

To explore connectivity for emotion-prioritization effect, we tested the interaction between networks using contrast [emotion (happy + sad) > neutral. The contrast with connectivity threshold *p* < 0.001 reveal one cluster (mass = 116.67, size = 6, p-FWE = 0.006). The cluster comprises positive connectivity between the DMN (MPFC) and three other networks: Frontoparietal (left PPC; *t*_(20)_ = 4.23, p-FWE = 0.048), Salience network (left RPFC; *t*_(20)_ = 4.62, p-FWE = 0.03) and DAN (bilateral FEF; *t*_(20)_ = 4.62, p-FWE = 0.03; *t*_(20)_ = 4.84, p-FWE = 0.034). There was also negative connectivity between the Salience network (left RPFC) and the Inferior Frontal Gyrus (*t*_(20)_ = −4.04, p-FWE = 0.05; Figure 3B). Systematically varying the connection threshold indicated that the cluster is formed by topologically focal connections (see details in Supplementary Table 5).

### Functional Connections Explaining Valence-Related Prioritization Effect

**Figure 4:**
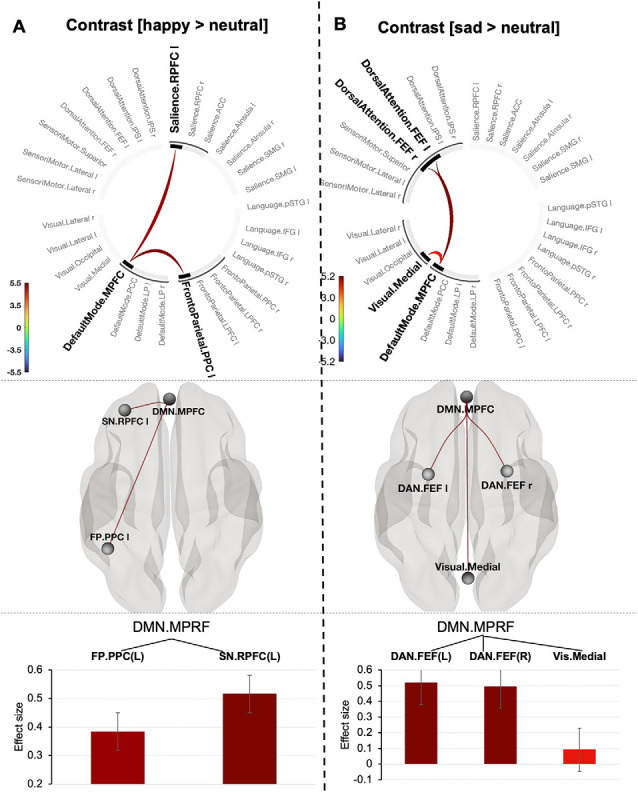
Connectogram of networks (top row), corresponding glass brain (middle row) and effect sizes of individual connections within a cluster (low row) for happy **(A)** and sad **(B)** prioritization effects [the connectivity threshold *p* < 0.001, cluster threshold p-FWE corrected (*p* < 0.05)]. Vertical color bars indicate T-test statistics for individual connections. Glass brain visualizes spatial location of connections comprising each component where a sphere represents the center of the corresponding network. The red and blue lines depict positive or negative correlations between networks. Effect sizes: the Y axis represents Pearson correlation values where the sign indicates the direction of the effect. Error bars represent standard deviations. The color of the effect size bars corresponds to the color of relevant connections in the connectogram.

The negative emotion prioritization effect defined by contrasting [sad > neutral] was associated with a significant network including the DMN (MPFC), Dorsal Attention network (bilateral frontal eye fields) and Visual Medial network (mass = 82.91, p-FWE = 0.013; size = 4; Figure 4A). Systematically varying the connectivity threshold indicated that this effect occurred only at a more conservative threshold (*p* < 0.004–0.0006; Supplementary Table 6).

A positive emotion-prioritization defined by contrast (happy > neutral) corresponded to one topological cluster comprising the MPFC of DMN network, Frontoparietal network (left posterior parietal cortex, PPC) and Salience network (left rostral prefrontal cortex, RPFC; *p* < 0.001, mass = 56.50, p-FWE = 0.034; size = 2; Figure 4B). Decreasing the connectivity threshold (*p* < 0.003) revealed a slightly larger component represented by additional connection between the DMN (MPFC) and Language network (posterior superior temporal gyrus, p-STG) yielding in total statistics with mass = 78.93, p-FWE = 0.032; size = 3). Further decreasing the “height” threshold revealed no significant results (Supplementary Table 7).

### Common and Distinct Network Connections Between Value-Based Reward and Emotion Prioritization Effects

**Figure 5:**
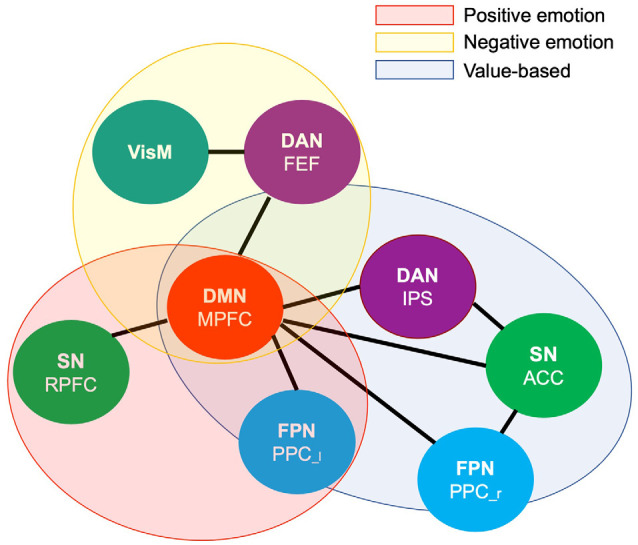
Venn diagram illustrating shared and distinct networks among value-based and emotional valence prioritization effects. DMN.MPFC (medial prefrontal node of the default mode network), FPN.PPC [left (l) and right (r) posterior cingulate cortex of the Frontoparietal network], DAN.FEF (bilateral frontal eye field of the Dorsal Attention network), DAN.IPS (intraparietal sulcus of the Dorsal Attention network), SN.RPFC (rostral prefrontal cortex of the Salience network), SN.ACC (anterior cingulate cortex of the salience network), VisM (visual medial network).

To summarize our main findings, mapping brain connectivity involved in the value-based and emotion prioritization effects indicated their overlap in the medial prefrontal node of the Default Mode network regardless of emotional valence. Positive emotion showed larger overlap with value-based processing by shared involvement of the medial prefrontal node of the Default Mode network and the left posterior parietal node of the Frontoparietal network compared to negative emotion with overlap only with respect to the medial prefrontal node of the Default Mode network (Figure 5).

## Discussion

The current study demonstrates that the brain forms distinct but partly overlapped components of interconnected networks for prioritizing value-based reward and emotion processing. The finding of partial overlap does not support the hypothesis that there may be an underlying system that is responsive to the basic properties of general affect (Park et al., 2019). However, the involvement of the MPFC node of the DMN in the value-based reward and emotion processing revealed by our NBS analysis may shed light on emotion regulation in the relationship between these behavioral drivers at the neural connection level.

### Common Connections Between Value-Based Reward and Emotion

The specific node in the MPFC of the DMN has been consistently associated with value-based decision making (Orsini et al., 2018), emotion regulation (Waugh et al., 2014) and self-related processes (Northoff, 2005; Sui et al., 2013; Hu et al., 2016) in a healthy population. Functional abnormalities within this node have been identified in virtually every psychiatric disorder with impaired processing of socially relevant cues such as reward, facial emotional expressions and self-relevance (Bittar and Labonté, 2021 for review). Converging evidence indicates that the MPFC node may function as a central hub in the brain (Sui, 2016), mediating processing of socially relevant information. This is supported by a substantial number of experimental human and laboratory animal studies demonstrating rich anatomical and functional connections between the MPFC and other cortical and subcortical areas (Riga et al., 2014). However, mapping the results of previous studies has been a challenging task due to a profusion of experimental and methodological approaches used in studies of reward and emotion processing (Oldham et al., 2018; Zhang et al., 2019; Flannery et al., 2020). We employed datasets with an identical experimental design allowing for tight control of factors triggering the value-based reward and emotion prioritization effects. The functional connectivity results associated with the DMN in our study provide an intriguing possibility to bridge two contrasting accounts of the neural overlap between value-based and emotion/valence processing (Chiew and Braver, 2011 for review). Particularly, the presence of the MPFC as a common node among clusters of neural networks involved in respective value-based, emotion and valence prioritization effects points toward its generic role in encoding the subjective value of incoming signals regardless of whether they are motivational or emotional (Sui and Gu, 2017). This can explain the similarities in behavioral performance for these effects, such as enhanced responses to stimuli with higher social value (i.e., higher reward value vs. lower reward value and stimuli containing emotional valence compared to neutral stimuli). This finding is in line with previous studies on reward processing (Peters and Büchel, 2010, for review) and lends further support to the proposal that the MPFC encodes emotion with a value signal similar to that for reward (Winecoff et al., 2013).

### Distinct Connections Between Reward and Positive (or Negative) Emotion

An interesting finding in our study is that value-based reward shares functional connections between the MPFC node of the DMN and the PPC node of the FPN with emotion processing, particularly with respect to positive emotion. However, the engagement of this connection emerges in the opposite direction: negative in the processing of value-based reward and positive in the processing of both happy and sad emotions. The PPC node of the FPN, in the dorsal angular gyrus, appears to be particularly sensitive to stimuli that can potentially become the focus of attention of the top-down attention system, such as reward incentives and emotion (Winecoff et al., 2013; Elward et al., 2015). Our results indicate that to facilitate value-based reward, the IPS node of DAN, a key node of salience processing (Sui et al., 2015a), forms a strong positive connection with the ACC node of the Salience network—a key region involved in the evaluation of engaging control to alter default actions in favor of better alternatives (Rushworth et al., 2011; Kolling et al., 2016). This finding resonates with a theoretical account proposing that maximizing potential reward relies on robust interactions between the attentional network and valuation networks, including the ACC and PPC (Pessoa and Engelmann, 2010). Previous studies demonstrated dynamic opposition between activation of the FPN and deactivation of the DMN and explained this relationship as transitions between rest and task-engaged states (Ossandón et al., 2011; Raichle, 2011; Chen et al., 2013; Sui et al., 2013). Disruptions in this dynamic opposition between these networks have been linked to attentional lapses and suboptimal performance in healthy subjects (Weissman et al., 2006; Prado and Weissman, 2011). We hypothesize that anticorrelation between the PPC and MPFC nodes observed here in the value-based reward task may play a role in regulating attentional control of the task. If steadfast, this hypothesis may advance our knowledge about neural mechanisms of attentional biases in addictions (Thomsen, 2015) and impairments in the ability to learn about reward in patients suffering from depression and schizophrenia.

#### Positive Emotion

The NBS analysis showed that positive emotion involves the RPFC node of SN and this node does not overlap with the value-based reward processing. Affective neuroscience provides evidence that the RPFC has been implicated in emotional regulation strategies (Viviani et al., 2010; Campbell-Sills et al., 2011; Mitchell, 2011) and appraisal or interpretation of emotion (Maier et al., 2012; Kreplin and Fairclough, 2013). However, recent clinical studies indicate that the role of the RPFC node of the Salience Network in emotion processing may be more complex than previously thought. For example, alterations in network functional connectivity associated with depressive symptoms were directly linked to dysfunctions in the RPFC (Fadel et al., 2021). It is worth mentioning that the left RPFC node in our study overlaps with previously reported “the dorsal nexus”—a so-called area in the prefrontal cortex that showed aberrant functional connectivity with the DMN, attention network and cognitive control network by hot-wiring them together in depression (Sheline et al., 2010). Moreover, some authors suggested that alterations in the dorsal nexus functional connectivity may serve as a biomarker for antidepressant effects (McCabe et al., 2011; Scheidegger et al., 2012). Exploring the precise role of the RPFC in the processing of positive emotion may provide a better understanding of its role in the development of mood disorders and clarify the Triple Network Model of psychopathology (Menon, 2011).

#### Negative Emotion

Our results suggest that two networks involved in the cluster supporting negative emotion biases (FEF and VisM) do not overlap with the reward processing. The role of the FEF node of the DAN is recognized in maintaining selective attention and providing attention-related feedback signals that regulate the quality of sensory processing in the visual cortex (Reynolds and Chelazzi, 2004; Squire et al., 2013). The importance of sensory sensitivity is underwritten by a recent trend in computational psychiatry that focuses on modeling sensory prediction errors within a predictive coding framework (Adams et al., 2013; Clark et al., 2018). However, despite the proposal that failure of regulating sensory sensitivity may be a key etiological factor in many mental health conditions with emotional dysfunctions (Acevedo et al., 2018 for review), the precise role of the FEF and VisM in the processing of negative emotional stimuli is yet to be established.

The NBS results in emotion processing showed that all nodes form positive connections with the MPFC of the DMN, indicating that the MPFC node plays a critical role in generating emotion biases. This finding is not surprising giving the wealth of studies reporting abnormal connectivity between the MPFC, FPN and SN in patients with mental health conditions. For example, hypoconnectivity between the MPFC and PPC nodes was observed in social anxiety disorder, where the reduction in this connection has been suggested as a possible neural basis for impairments in the perception of socially relevant emotional states (Qiu et al., 2011). Furthermore, aberrant connectivity between the MPFC and FPN/SN nodes has been found in patients with major depression (Zheng et al., 2015; Fadel et al., 2021), schizophrenia (Manoliu et al., 2014), bipolar disorder (Lopez-Larson et al., 2017; Wang et al., 2020) and psychosis (Wotruba et al., 2014). However, previous research provided inconsistent evidence of the role that the MPFC plays in the processing of emotional valence. For example, some studies proposed that positive and negative affective processing exhibit dissociable functional hubs outside the MPFC (Zhang et al., 2015), while others suggested the MPFC as a functional hub for both positive and negative emotions (Yang et al., 2018) or positive only (Lindquist et al., 2016; Machado and Cantilino, 2017). The results of our study indicate that the MPFC may serve as a connectivity hub for both positive and negative emotions with the differences in functional connections between the MPFC and FPN, DAN and SN, indicating the mainstream for processing happy and sad emotions.

## Limitations

It is important to note that our findings do not represent causal relationships due to the nature of the analyses used. That is, the clusters of interconnected networks do not unveil the directions of their interactions. In addition, our findings need to be validated on separate and larger datasets. Although the NBS method overcomes some limitations of graph analytical approaches (Fornito et al., 2013), network-based data analysis does not have a notion of sample size. Unlike standard statistical inferences in activation studies (i.e., typically, t-test or parametric analysis of variance), NBS and similar methods use second-order metrics, multi-threshold and permutation techniques, non-parametric statistics that are more robust than the standard approaches (Braun et al., 2012). This calls for developing methods for calculating sample size in network-based research. Until then, validation of findings on separate datasets can be used to estimate the reliability of these findings. Third, studies on brain networks use different parcellation scales and nomenclature (Uddin et al., 2019), which limits comparisons between the results of our study and previous work.

## Conclusion

Value-based reward and emotion prioritization effects overlap in the MPFC node of the DMN regardless of emotional valence. Positive emotion processing shares the MPFC node and the PPC node of the FPN, with value-based reward processing showing a larger overlap compared to negative emotion processing. Value-based reward and emotion biases differentially involve nodes of the DMN, FPN, DAN, and SN. These findings support the alternative hypothesis that the brain forms partly overlapping but different components of interconnected networks such as DMN, DAN, SN, and FPN to prioritize value-based reward and emotional information. Facilitating our understanding of how information flows between these networks will require shifting the debate about overlapping neural substrates for value-based reward and emotion processing toward mapping the relationship between these networks, advancing our understanding behavioral drivers in relation to normal and psychopathological processes.

## Data Availability Statement

The original contributions presented in the study are included in the article/Supplementary Material, further inquiries can be directed to the corresponding author.

## Ethics Statement

The studies involving human participants were reviewed and approved by Central University of Oxford Research Ethics Committee (CUREC). The patients/participants provided their written informed consent to participate in this study.

## Author Contributions

AY and JS were involved in the design, data analysis, and conceptualization of the study. AY was the main writer of the manuscript. TD-S was involved in data preprocessing, data quality analysis, and editing. DY, AG, and BC were involved in the revision of the results, reviewing of the manuscript, and editing. All authors contributed to the article and approved the submitted version.

## Conflict of Interest

The authors declare that the research was conducted in the absence of any commercial or financial relationships that could be construed as a potential conflict of interest.

## Publisher’s Note

All claims expressed in this article are solely those of the authors and do not necessarily represent those of their affiliated organizations, or those of the publisher, the editors and the reviewers. Any product that may be evaluated in this article, or claim that may be made by its manufacturer, is not guaranteed or endorsed by the publisher.

## Footnotes

^1^ http://www.neurobs.com

^2^ www.fil.ion.ucl.ac.uk/spm

## References

[B1] Acevedo B. Aron E. Pospos S. Jessen D. (2018). The functional highly sensitive brain: a review of the brain circuits underlying sensory processing sensitivity and seemingly related disorders. Philos. Trans. R. Soc. Lond. B Biol. sci. 373:20170161. 10.1098/rstb.2017.0161 29483346 PMC5832686

[B2] Adams R. A. Stephan K. E. Brown H. R. Frith C. D. Friston K. J. (2013). The computational anatomy of psychosis. Front. Psychiatry 4:47. 10.3389/fpsyt.2013.00047 23750138 PMC3667557

[B3] Alegria A. A. Radua J. Rubia K. (2016). Meta-analysis of fMRI studies of disruptive behavior disorders. Am. J. Psychiatry 173, 1119–1130. 10.1176/appi.ajp.2016.15081089 27523497

[B5] Anderson B. A. Laurent P. A. Yantis S. (2011). Value-driven attentional capture. Proc. Natl. Acad. Sci. U S A 108, 10367–10371. 10.1073/pnas.1104047108 21646524 PMC3121816

[B4] Anderson B. A. Yantis S. (2013). Persistence of value-driven attentional capture. J. Exp. Psychol. Hum. Percept. Perform. 39, 6–9. 10.1037/a0030860 23181684 PMC3989924

[B6] Ashburner J. Friston K. J. (2005). Unified segmentation. Neuroimage 26, 839–851. 10.1016/j.neuroimage.2005.02.018 15955494

[B7] Barkus E. Badcock J. C. (2019). A transdiagnostic perspective on social anhedonia. Front. Psychiatry 10:216. 10.3389/fpsyt.2019.00216 31105596 PMC6491888

[B8] Bartra O. McGuire J. T. Kable J. W. (2013). The valuation system: a coordinate-based meta-analysis of BOLD fMRI experiments examining neural correlates of subjective value. Neuroimage 76, 412–427. 10.1016/j.neuroimage.2013.02.063 23507394 PMC3756836

[B9] Bassett D. S. Sporns O. (2017). Network neuroscience. Nat. Neurosci. 20, 353–364. 10.1038/nn.4502 28230844 PMC5485642

[B10] Behzadi Y. Restom K. Liau J. Liu T. T. (2007). A component based noise correction method (CompCor) for BOLD and perfusion based fMRI. Neuroimage 37, 90–101. 10.1016/j.neuroimage.2007.04.042 17560126 PMC2214855

[B11] Birn R. M. Cornejo M. D. Molloy E. K. Patriat R. Meier T. B. Kirk G. R.. (2014). The influence of physiological noise correction on test-retest reliability of resting-state functional connectivity. Brain Connect. 4, 511–522. 10.1089/brain.2014.0284 25112809 PMC4146390

[B12] Bittar T. P. Labonté B. (2021). Functional contribution of the medial prefrontal circuitry in major depressive disorder and stress-induced depressive-like behaviors. Front. Behav. Neurosci. 15:699592. 10.3389/fnbeh.2021.699592 34234655 PMC8257081

[B13] Brandl F. Le Houcq Corbi Z. Mulej Bratec S. Sorg C. (2019). Cognitive reward control recruits medial and lateral frontal cortices, which are also involved in cognitive emotion regulation: a coordinate-based meta-analysis of fMRI studies. Neuroimage 200, 659–673. 10.1016/j.neuroimage.2019.07.008 31280010

[B14] Braun U. Plichta M. M. Esslinger C. Sauer C. Haddad L. Grimm O.. (2012). Test-retest reliability of resting-state connectivity network characteristics using fMRI and graph theoretical measures. Neuroimage 59, 1404–1412. 10.1016/j.neuroimage.2011.08.044 21888983

[B15] Buck R. (2000). Conceptualizing motivation and emotion. Behav. Brain Sci. 23, 195–196. 10.1017/S0140525X00262420

[B16] Caballero-Gaudes C. Reynolds R. C. (2017). Methods for cleaning the BOLD fMRI signal. Neuroimage 154, 128–149. 10.1016/j.neuroimage.2016.12.018 27956209 PMC5466511

[B17] Campbell-Sills L. Simmons A. N. Lovero K. L. Rochlin A. A. Paulus M. P. Stein M. B. (2011). Functioning of neural systems supporting emotion regulation in anxiety-prone individuals. NeuroImage 54, 689–696. 10.1016/j.neuroimage.2010.07.041 20673804 PMC2962684

[B18] Chai X. J. Castañón A. N. Ongür D. Whitfield-Gabrieli S. (2012). Anticorrelations in resting state networks without global signal regression. Neuroimage 59, 1420–1428. 10.1016/j.neuroimage.2011.08.048 21889994 PMC3230748

[B19] Chen A. C. Oathes D. J. Chang C. Bradley T. Zhou Z. W. Williams L. M.. (2013). Causal interactions between fronto-parietal central executive and default-mode networks in humans. Proc. Natl. Acad. Sci. U S A 110, 19944–19949. 10.1073/pnas.1311772110 24248372 PMC3856839

[B20] Chiew K. S. Braver T. S. (2011). Positive affect versus reward: emotional and motivational influences on cognitive control. Front. Psychol. 2:279. 10.3389/fpsyg.2011.00279 22022318 PMC3196882

[B21] Ciric R. Wolf D. H. Power J. D. Roalf D. R. Baum G. L. Ruparel K.. (2017). Benchmarking of participant-level confound regression strategies for the control of motion artifact in studies of functional connectivity. Neuroimage 154, 174–187. 10.1016/j.neuroimage.2017.03.020 28302591 PMC5483393

[B22] Clark J. E. Watson S. Friston K. J. (2018). What is mood? A computational perspective. Psychol. Med. 48, 2277–2284. 10.1017/S0033291718000430 29478431 PMC6340107

[B23] Cole M. W. Bassett D. S. Power J. D. Braver T. S. Petersen S. E. (2014). Intrinsic and task-evoked network architectures of the human brain. Neuron 83, 238–251. 10.1016/j.neuron.2014.05.014 24991964 PMC4082806

[B24] Cole M. W. Ito T. Bassett D. S. Schultz D. H. (2016). Activity flow over resting-state networks shapes cognitive task activations. Nat. Neurosci. 19, 1718–1726. 10.1038/nn.4406 27723746 PMC5127712

[B25] Cole M. W. Ito T. Schultz D. Mill R. Chen R. Cocuzza C. (2019). Task activations produce spurious but systematic inflation of task functional connectivity estimates. Neuroimage 189, 1–18. 10.1016/j.neuroimage.2018.12.054 30597260 PMC6422749

[B26] Cromwell H. C. Abe N. Barrett K. C. Caldwell-Harris C. Gendolla G. Koncz R.. (2020). Mapping the interconnected neural systems underlying motivation and emotion: a key step toward understanding the human affectome. Neurosci. Biobehav. Rev. 113, 204–226. 10.1016/j.neubiorev.2020.02.032 32126241

[B27] Elward R. L. Vilberg K. L. Rugg M. D. (2015). Motivated memories: effects of reward and recollection in the core recollection network and beyond. Cereb. Cortex 25, 3159–3166. 10.1093/cercor/bhu109 24872520 PMC4537449

[B28] Fadel E. Boeker H. Gaertner M. Richter A. Kleim B. Seifritz E.. (2021). Differential alterations in resting state functional connectivity associated with depressive symptoms and early life adversity. Brain Sci. 11:591. 10.3390/brainsci11050591 34063232 PMC8147478

[B29] Fiori M. Shuman V. (2017). The joint contribution of activation and inhibition in moderating carryover effects of anger on social judgment. Front. Psychol. 8:1435. 10.3389/fpsyg.2017.01435 28993743 PMC5622303

[B30] Flannery J. S. Riedel M. C. Bottenhorn K. L. Poudel R. Salo T. Hill-Bowen L. D.. (2020). Meta-analytic clustering dissociates brain activity and behavior profiles across reward processing paradigms. Cogn. Affect. Behav. Neurosci. 20, 215–235. 10.3758/s13415-019-00763-7 31872334 PMC7117996

[B31] Fornito A. Zalesky A. Breakspear M. (2013). Graph analysis of the human connectome: promise, progress and pitfalls. NeuroImage 80, 426–444. 10.1016/j.neuroimage.2013.04.087 23643999

[B32] Fornito A. Zalesky A. Breakspear M. (2015). The connectomics of brain disorders. Nat. Rev. Neurosci. 16, 159–172. 10.1038/nrn3901 25697159

[B33] Fox E. (2018). Perspectives from affective science on understanding the nature of emotion. Brain Neurosci. Adv. 2:2398212818812628. 10.1177/2398212818812628 32166161 PMC7058241

[B34] Fox E. Russo R. Dutton K. (2002). Attentional bias for threat: evidence for delayed disengagement from emotional faces. Cogn. Emot. 16, 355–379. 10.1080/02699930143000527 18273395 PMC2241753

[B35] Grill F. Nyberg L. Rieckmann A. (2021). Neural correlates of reward processing: functional dissociation of two components within the ventral striatum. Brain Behav. 11:e01987. 10.1002/brb3.1987 33300306 PMC7882172

[B36] Hoemann K. Devlin M. Barrett L. F. (2020). Comment: emotions are abstract, conceptual categories that are learned by a predicting brain. Emot. Rev. 12, 253–255. 10.1177/1754073919897296

[B37] Hu C. Di X. Eickhoff S. B. Zhang M. Peng K. Guo H.. (2016). Distinct and common aspects of physical and psychological self-representation in the brain: a meta-analysis of self-bias in facial and self-referential judgements. Neurosci. Biobehav. Rev. 61, 197–207. 10.1016/j.neubiorev.2015.12.003 26695384

[B39] Jauhar S. Fortea L. Solanes A. Albajes-Eizagirre A. McKenna P. J. Radua J. (2021). Brain activations associated with anticipation and delivery of monetary reward: a systematic review and meta-analysis of fMRI studies. PLoS One 16:e0255292. 10.1371/journal.pone.0255292 34351957 PMC8341642

[B40] Jiang X. Zhao L. Liu H. Guo L. Kendrick K. M. Liu T. (2018). A cortical folding pattern-guided model of intrinsic functional brain networks in emotion processing. Front. Neurosci. 12:575. 10.3389/fnins.2018.00575 30186102 PMC6110906

[B41] Kolling N. Wittmann M. K. Behrens T. E. Boorman E. D. Mars R. B. Rushworth M. F. (2016). Value, search, persistence and model updating in anterior cingulate cortex. Nat. Neurosci. 19, 1280–1285. 10.1038/nn.4382 27669988 PMC7116891

[B42] Kragel P. A. LaBar K. S. (2016). Decoding the nature of emotion in the brain. Trends Cogn. Sci. 20, 444–455. 10.1016/j.tics.2016.03.011 27133227 PMC4875847

[B43] Kreplin U. Fairclough S. H. (2013). Activation of the rostromedial prefrontal cortex during the experience of positive emotion in the context of esthetic experience. An fNIRS study. Front. Hum. Neurosci. 7:879. 10.3389/fnhum.2013.00879 24391572 PMC3868912

[B44] Laming D. (2000). On the behavioural interpretation of neurophysiological observation. Behav. Brain Sci. 23:209. 10.1017/S0140525X00392421

[B45] Lang P. J. Bradley M. M. (2008). “Appetitive and defensive motivation is the substrate of emotion,” in Handbook of Approach and Avoidance Motivation, ed Elliot A. J. (New York; Hove: Psychology Press, Taylor and Francis Group), 51–65.

[B46] Lindquist K. A. Satpute A. B. Wager T. D. Weber J. Barrett L. F. (2016). The brain basis of positive and negative affect: evidence from a meta-analysis of the human neuroimaging literature. Cereb. Cortex 26, 1910–1922. 10.1093/cercor/bhv001 25631056 PMC4830281

[B83] Lin S.-Y. Lee C.-C. Chen Y.-S. Kuo L.-W. (2019). Investigation of functional brain network reconfiguration during vocal emotional processing using graph-theoretical analysis. Soc. Cogn. Affect. Neurosci. 14, 529–538. 10.1093/scan/nsz025 31157395 PMC6545541

[B48] Lopez-Larson M. P. Shah L. M. Weeks H. R. King J. B. Mallik A. K. Yurgelun-Todd D. A.. (2017). Abnormal functional connectivity between default and salience networks in pediatric bipolar disorder. Biol. Psychiatry Cogn. Neurosci. Neuroimaging 2, 85–93. 10.1016/j.bpsc.2016.10.001 29560889 PMC6422527

[B49] Machado L. Cantilino A. (2017). A systematic review of the neural correlates of positive emotions. Braz. J. Psychiatry 39, 172–179. 10.1590/1516-4446-2016-1988 27901215 PMC7111451

[B50] Maier S. Szalkowski A. Kamphausen S. Perlov E. Feige B. Blechert J.. (2012). Clarifying the role of the rostral dmPFC/dACC in fear/anxiety: learning, appraisal or expression? PLoS One 7:e50120. 10.1371/journal.pone.0050120 23189183 PMC3506550

[B51] Manoliu A. Riedl V. Zherdin A. Mühlau M. Schwerthöffer D. Scherr M.. (2014). Aberrant dependence of default mode/central executive network interactions on anterior insular salience network activity in schizophrenia. Schizophr. Bull. 40, 428–437. 10.1093/schbul/sbt037 23519021 PMC3932085

[B52] McCabe C. Mishor Z. Filippini N. Cowen P. J. Taylor M. J. Harmer C. J. (2011). SSRI administration reduces resting state functional connectivity in dorso-medial prefrontal cortex. Mol. Psychiatry 16, 592–594. 10.1038/mp.2010.138 21263442

[B53] Menon V. (2011). Large-scale brain networks and psychopathology: a unifying triple network model. Trends Cogn. Sci. 15, 483–506. 10.1016/j.tics.2011.08.003 21908230

[B54] Mitchell D. G. V. (2011). The nexus between decision making and emotion regulation: a review of convergent neurocognitive substrates. Behav. Brain Res. 217, 215–231. 10.1016/j.bbr.2010.10.030 21055420

[B55] Murphy K. Birn R. M. Handwerker D. A. Jones T. B. Bandettini P. A. (2009). The impact of global signal regression on resting state correlations: are anti-correlated networks introduced? NeuroImage 44, 893–905. 10.1016/j.neuroimage.2008.09.036 18976716 PMC2750906

[B56] Northoff G. (2005). Emotional-cognitive integration, the self and cortical midline structures. Behav. Brain Sci. 28, 211–212. 10.1017/S0140525X05400047

[B57] Oldham S. Murawski C. Fornito A. Youssef G. Yücel M. Lorenzetti V. (2018). The anticipation and outcome phases of reward and loss processing: a neuroimaging meta-analysis of the monetary incentive delay task. Hum. Brain Mapp. 39, 3398–3418. 10.1002/hbm.24184 29696725 PMC6055646

[B58] Ono Y. Taniguchi Y. (2017). Attentional capture by emotional stimuli: manipulation of emotional valence by the sample pre-rating method. Jpn. Psychol. Res. 59, 26–34. 10.1111/jpr.12142

[B59] Orsini C. A. Heshmati S. C. Garman T. S. Wall S. C. Bizon J. L. Setlow B. (2018). Contributions of medial prefrontal cortex to decision making involving risk of punishment. Neuropharmacology 139, 205–216. 10.1016/j.neuropharm.2018.07.018 30009836 PMC6108435

[B60] Ossandón T. Jerbi K. Vidal J. R. Bayle D. J. Henaff M. A. Jung J.. (2011). Transient suppression of broadband gamma power in the default-mode network is correlated with task complexity and subject performance. J. Neurosci. 31, 14521–14530. 10.1523/JNEUROSCI.2483-11.2011 21994368 PMC6703400

[B61] Palminteri S. Khamassi M. Joffily M. Coricelli G. (2015). Contextual modulation of value signals in reward and punishment learning. Nat. Commun. 6:8096. 10.1038/ncomms9096 26302782 PMC4560823

[B62] Pan J. Zhan L. Hu C. Yang J. Wang C. Gu L.. (2018). Emotion regulation and complex brain networks: association between expressive suppression and efficiency in the fronto-parietal network and default-mode network. Front. Hum. Neurosci. 12:70. 10.3389/fnhum.2018.00070 29662443 PMC5890121

[B63] Panksepp J. (1998). Affective Neuroscience: The Foundations of Human and Animal Emotions. New York: Oxford University Press.

[B64] Park H. R. P. Kostandyan M. Boehler C. N. Krebs R. M. (2019). Winning smiles: signalling reward by overlapping and non-overlapping emotional valence differentially affects performance and neural activity. Neuropsychologia 122, 28–37. 10.1016/j.neuropsychologia.2018.11.018 30521814

[B65] Park H. P. R. Kostandyan M. Boehler C. N. Krebs R. M. (2018). Smiling faces and cash bonuses: exploring common affective coding across positive and negative emotional and motivational stimuli using fMRI. Cogn. Affect. Behav. Neurosci. 18, 550–563. 10.3758/s13415-018-0587-3 29644568

[B66] Pessoa L. (2009). How do emotion and motivation direct executive control? Trends Cogn. Sci. 13, 160–166. 10.1016/j.tics.2009.01.006 19285913 PMC2773442

[B67] Pessoa L. Engelmann J. B. (2010). Embedding reward signals into perception and cognition. Front. Neurosci. 4:17. 10.3389/fnins.2010.00017 20859524 PMC2940450

[B68] Peters J. Büchel C. (2010). Neural representations of subjective reward value. Behav. Brain Res. 213, 135–141. 10.1016/j.bbr.2010.04.031 20420859

[B69] Power J. D. Mitra A. Laumann T. O. Snyder A. Z. Schlaggar B. L. Petersen S. E. (2014). Methods to detect, characterize and remove motion artifact in resting state fMRI. Neuroimage 84, 320–341. 10.1016/j.neuroimage.2013.08.048 23994314 PMC3849338

[B70] Prado J. Weissman D. H. (2011). Heightened interactions between a key default-mode region and a key task-positive region are linked to suboptimal current performance but to enhanced future performance. Neuroimage 56, 2276–2282. 10.1016/j.neuroimage.2011.03.048 21440073

[B71] Qiu C. Liao W. Ding J. Feng Y. Zhu C. Nie X.. (2011). Regional homogeneity changes in social anxiety disorder: a resting-state fMRI study. Psychiatry Res. 194, 47–53. 10.1016/j.pscychresns.2011.01.010 21831605

[B72] Raichle M. E. (2011). The restless brain. Brain Connect. 1, 3–12. 10.1089/brain.2011.0019 22432951 PMC3621343

[B73] Reynolds J. H. Chelazzi L. (2004). Attentional modulation of visual processing. Annu. Rev. Neurosci. 27, 611–647. 10.1146/annurev.neuro.26.041002.131039 15217345

[B74] Riga D. Matos M. R. Glas A. Smit A. B. Spijker S. Van den Oever M. C. (2014). Optogenetic dissection of medial prefrontal cortex circuitry. Front. Syst. Neurosci. 8:230. 10.3389/fnsys.2014.00230 25538574 PMC4260491

[B75] Rushworth M. F. S. Noonan M. P. Boorman E. D. Walton M. E. Behrens T. E. (2011). Frontal cortex and reward-guided learning and decision-making. Neuron 70, 1054–1069. 10.1016/j.neuron.2011.05.014 21689594

[B76] Ryan F. Skandali N. (2016). Editorial: reward processing in motivational and affective disorders. Front. Psychol. 7:1288. 10.3389/fpsyg.2016.01288 27625619 PMC5003886

[B77] Sabharwal A. Szekely A. Kotov R. Mukherjee P. Leung H. C. Barch D. M.. (2016). Transdiagnostic neural markers of emotion-cognition interaction in psychotic disorders. J. Abnorm. Psychol. 125, 907–922. 10.1037/abn0000196 27618279 PMC5576592

[B78] Sander D. Nummenmaa L. (2021). Reward and emotion: an affective neuroscience approach. Curr. Opin. Behav. Sci. 39, 161–167. 10.1016/j.cobeha.2021.03.016

[B79] Scalabrini A. Mucci C. Esposito R. Damiani S. Northoff G. (2020). Dissociation as a disorder of integration - On the footsteps of Pierre Janet. Prog. Neuropsychopharmacol. Biol. Psychiatry 101:109928. 10.1016/j.pnpbp.2020.109928 32194203

[B80] Scheidegger M. Walter M. Lehmann M. Metzger C. Grimm S. Boeker H.. (2012). Ketamine decreases resting state functional network connectivity in healthy subjects: implications for antidepressant drug action. PLoS One 7:e44799. 10.1371/journal.pone.0044799 23049758 PMC3461985

[B81] Sheline Y. I. Price J. L. Yan Z. Mintun M. A. (2010). Resting-state functional MRI in depression unmasks increased connectivity between networks *via* the dorsal nexus. Proc. Natl. Acad. Sci. U S A 107, 11020–11025. 10.1073/pnas.1000446107 20534464 PMC2890754

[B82] Shi G. Li X. Zhu Y. Shang R. Sun Y. Guo H.. (2021). The divided brain: functional brain asymmetry underlying self-construal. NeuroImage 240:118382. 10.1016/j.neuroimage.2021.118382 34252524

[B84] Smith S. M. Fox P. T. Miller K. L. Glahn D. C. Fox P. M. Mackay C. E.. (2009). Correspondence of the brain’s functional architecture during activation and rest. Proc. Natl. Acad. Sci. U S A 106, 13040–13045. 10.1073/pnas.0905267106 19620724 PMC2722273

[B85] Squire R. F. Noudoost B. Schafer R. J. Moore T. (2013). Prefrontal contributions to visual selective attention. Annu. Rev. Neurosci. 36, 451–466. 10.1146/annurev-neuro-062111-150439 23841841

[B86] Stolte M. Humphreys G. Yankouskaya A. Sui J. (2017). Dissociating biases towards the self and positive emotion. Q. J. Exp. Psychol. (Hove) 70, 1011–1022. 10.1080/17470218.2015.1101477 26444388

[B87] Stolte M. Spence C. Barutchu A. (2021). Multisensory perceptual biases for social and reward associations. Front. Psychol. 12:640684. 10.3389/fpsyg.2021.640684 33776865 PMC7990908

[B88] Sui J. (2016). Self-reference acts as a golden thread in binding. Trends Cogn. Sci. 20, 482–483. 10.1016/j.tics.2016.04.005 27315761 PMC6029663

[B89] Sui J. Gu X. (2017). Self as object: emerging trends in self research. Trends Neurosci. 40, 643–653. 10.1016/j.tins.2017.09.002 28988827

[B90] Sui J. He X. Humphreys G. W. (2012). Perceptual effects of social salience: evidence from self-prioritization effects on perceptual matching. J. Exp. Psychol. Hum. Percept. Perform. 38, 1105–1117. 10.1037/a0029792 22963229

[B91] Sui J. Liu M. Mevorach C. Humphreys G. W. (2015a). The salient self: the left intra-parietal sulcus responds to social as well as perceptual-salience after self-association. Cereb. Cortex 25, 1060–1068. 10.1093/cercor/bht302 24165832

[B93] Sui J. Yankouskaya A. Humphreys G. W. (2015b). Super-capacity me! Super-capacity and violations of race independence for self- but not for reward-associated stimuli. J. Exp. Psychol. Hum. Percept. Perform. 41, 441–452. 10.1037/a0038288 25602970

[B92] Sui J. Rotshtein P. Humphreys G. W. (2013). Coupling social attention to the self forms a network for personal significance. Proc. Natl. Acad. Sci. U S A 110, 7607–7612. 10.1073/pnas.1221862110 23610386 PMC3651422

[B94] Thomsen K. R. (2015). Measuring anhedonia: impaired ability to pursue, experience and learn about reward. Front. Psychol. 6:1409. 10.3389/fpsyg.2015.01409 26441781 PMC4585007

[B95] Uddin L. Q. Yeo B. T. T. Spreng R. N. (2019). Towards a universal taxonomy of macro-scale functional human brain networks. Brain Topogr. 32, 926–942. 10.1007/s10548-019-00744-6 31707621 PMC7325607

[B96] Van Dijk K. R. A. Sabuncu M. R. Buckner R. L. (2012). The influence of head motion on intrinsic functional connectivity MRI. NeuroImage 59, 431–438. 10.1016/j.neuroimage.2011.07.044 21810475 PMC3683830

[B97] Vartak D. Jeurissen D. Self M. W. Roelfsema P. R. (2017). The influence of attention and reward on the learning of stimulus-response associations. Sci. Rep. 7:9036. 10.1038/s41598-017-08200-w 28831043 PMC5567207

[B98] Viviani R. Lo H. Sim E. J. Beschoner P. Stingl J. C. Horn A. B. (2010). The neural substrate of positive bias in spontaneous emotional processing. PLoS One 5:e15454. 10.1371/journal.pone.0015454 21079747 PMC2975711

[B99] Wang J. Wang Y. Huang H. Jia Y. Zheng S. Zhong S.. (2020). Abnormal dynamic functional network connectivity in unmedicated bipolar and major depressive disorders based on the triple-network model. Psychol. Med. 50, 465–474. 10.1017/S003329171900028X 30868989

[B100] Watson P. Pearson D. Theeuwes J. Most S. B. Le Pelley M. E. (2020). Delayed disengagement of attention from distractors signalling reward. Cognition 195:104125. 10.1016/j.cognition.2019.104125 31751815

[B101] Waugh C. E. Lemus M. G. Gotlib I. H. (2014). The role of the medial frontal cortex in the maintenance of emotional states. Soc. Cogn. Affect. Neurosci. 9, 2001–2009. 10.1093/scan/nsu011 24493835 PMC4249480

[B102] Weissman D. H. Roberts K. C. Visscher K. M. Woldorff M. G. (2006). The neural bases of momentary lapses in attention. Nat. Neurosci. 9, 971–978. 10.1038/nn1727 16767087

[B103] Whitton A. E. Treadway M. T. Pizzagalli D. A. (2015). Reward processing dysfunction in major depression, bipolar disorder and schizophrenia. Curr. Opin. Psychiatry 28, 7–12. 10.1097/YCO.0000000000000122 25415499 PMC4277233

[B104] Winecoff A. Clithero J. A. Carter R. M. Bergman S. R. Wang L. Huettel S. A. (2013). Ventromedial prefrontal cortex encodes emotional value. J. Neurosci. 33, 11032–11039. 10.1523/JNEUROSCI.4317-12.2013 23825408 PMC3718369

[B105] Wotruba D. Michels L. Buechler R. Metzler S. Theodoridou A. Gerstenberg M.. (2014). Aberrant coupling within and across the default mode, task-positive and salience network in subjects at risk for psychosis. Schizophr. Bull. 40, 1095–1104. 10.1093/schbul/sbt161 24243441 PMC4133671

[B106] Yang X. Garcia K. M. Jung Y. Whitlow C. T. McRae K. Waugh C. E. (2018). vmPFC activation during a stressor predicts positive emotions during stress recovery. Soc. Cogn. Affect. Neurosci. 13, 256–268. 10.1093/scan/nsy012 29462404 PMC5836276

[B107] Yankouskaya A. Sui J. (2021). Self-positivity or self-negativity as a function of the medial prefrontal cortex. Brain Sci. 11:264. 10.3390/brainsci11020264 33669682 PMC7922957

[B109] Yankouskaya A. Humphreys G. Stolte M. Stokes M. Moradi Z. Sui J. (2017). An anterior-posterior axis within the ventromedial prefrontal cortex separates self and reward. Soc. Cogn. Affect. Neurosci. 12, 1859–1868. 10.1093/scan/nsx112 29040796 PMC5716107

[B110] Young C. B. Chen T. Nusslock R. Keller J. Schatzberg A. F. Menon V. (2016). Anhedonia and general distress show dissociable ventromedial prefrontal cortex connectivity in major depressive disorder. Transl. Psychiatry 6:e810. 10.1038/tp.2016.80 27187232 PMC5070048

[B111] Zalesky A. Fornito A. Bullmore E. T. (2010). Network-based statistic: identifying differences in brain networks. NeuroImage 53, 1197–1207. 10.1016/j.neuroimage.2010.06.041 20600983

[B113] Zhang W. Li H. Pan X. (2015). Positive and negative affective processing exhibit dissociable functional hubs during the viewing of affective pictures. Hum. Brain Mapp. 36, 415–426. 10.1002/hbm.22636 25220389 PMC6869282

[B112] Zhang B. Lin P. Shi H. Öngür D. Auerbach R. P. Wang X.. (2016). Mapping anhedonia-specific dysfunction in a transdiagnostic approach: an ALE meta-analysis. Brain Imaging Behav. 10, 920–939. 10.1007/s11682-015-9457-6 26487590 PMC4838562

[B114] Zhang Y. Padmanabhan A. Gross J. J. Menon V. (2019). Development of human emotion circuits investigated using a big-data analytic approach: stability, reliability and robustness. J. Neurosci. 39, 7155–7172. 10.1523/JNEUROSCI.0220-19.2019 31332001 PMC6733549

[B115] Zheng H. Xu L. Xie F. Guo X. Zhang J. Yao L.. (2015). The altered triple networks interaction in depression under resting state based on graph theory. Biomed. Res. Int. 2015:386326. 10.1155/2015/386326 26180798 PMC4477135

[B116] Zhu Y. Li X. Sun Y. Wang H. Guo H. Sui J. (2021). “Investigating neural substrates of individual independence and interdependence orientations *via* efficiency-based dynamic functional connectivity: a machine learning approach,” in IEEE Transactions on Cognitive and Developmental Systems. 10.1109/TCDS.2021.3101643

