# Supplementary material for: Neural Connectivity Underlying Reward and Emotion-Related Processing: Evidence From a Large-Scale Network Analysis
Source: Front Syst Neurosci. 2022 Apr 7;16:833625. doi: 10.3389/fnsys.2022.833625 (PMC9033203; doi:10.3389/fnsys.2022.833625)
Supplement: Supplementary file 1 [file Data_Sheet_1.PDF]

Supplementary Materials

Neural connectivity underlying reward and emotion-related processing: Evidence from a large-scale network analysis

Supplementary Table 1. Summary of datasets and behavioural results.

Dataset 1 *	Dataset 2 **
Participants	
In both studies, participants reported no use of psychotropic medications or past diagnoses for psychiatric, neurological disorders and have normal or corrected-to-normal vision size.	
Twenty-one young, healthy adults aged between 21-26 (10 males, age M=23.6, SD =2.8). As a part of the pre-screening procedure, participants performed Mood and Anxiety Symptom Questionnaire (MASQ), a 77-items self-report questionnaire that assesses depressive, anxious and mixed symptomatology. Only participants with low scores on each of 5 subscales were invited to the scanning session.	In the original study, we reported results from sixteen healthy volunteers. In the present study, we added data from three participants more who completed the value-based task yielding in total sample size of nineteen individuals aged between 22-34 (9 males, age M = 25.8, SD = 7.31). For each correct answer in the value-based task, participants received a reward of 2% of the amount of money displayed on a given trial.
Stimuli	
Dataset 1: six geometric shapes (circle, hexagon, square, rectangle, diamond and triangle) were randomly assigned to three conditions (happy, sad, neutral). A stimulus display contained a fixation cross (0.7°×0.7°) on the center of the screen with a shape (covering 3.5° × 3.5° of visual angle) and label (or a schematic face) covering 1.76°/ 2.52° × 1.76° (3.5° × 3.5°) of visual angle on either side of fixation. The distance between the shape and the label (or a schematic face) was 10 degrees. Left-right presentations of the shapes and labels/schematic faces were counterbalanced across trials. Each trial started with a fixation cross for 200 ms, followed by the stimulus display for 100 ms and a blank interval which remained for 1000 ms or until the participant responded. Trials were separated by a jittered interstimulus interval (ranging between 2500-6000 ms).<br><br>*Dataset 2*: Four geometric shapes (circle, hexagon, square, and triangle) were randomly assigned across participants to two value-based conditions (high reward, low reward). The stimulus display contained a fixation cross (0.8°×0.8°) at the center of the screen with a shape (3.8°×3.8°) and label on either side of fixation. The distance between shape and label was 10°. Presentations of the shapes and labels were counterbalanced across trials. Each trial started with a fixation cross for 200 ms, followed by the stimulus display for 100 ms and a blank interval which remained for 1000 ms. or until the participant responded. Trials were separated by a jittered interstimulus interval (ranging between 2500-6000 ms).	
Trial number	
Five runs of 72 trials in each task	Four runs of 48 trials

* Yankouskaya, A., & Sui, J. (2021). Self-Positivity or Self-Negativity as a Function of the Medial Prefrontal Cortex. *Brain sciences*, *11*(2), 264. https://doi.org/10.3390/brainsci11020264

**Yankouskaya, A., Humphreys, G., Stolte, M., Stokes, M., Moradi, Z., & Sui, J. (2017). An anterior-posterior axis within the ventromedial prefrontal cortex separates self and reward. *Social cognitive and affective neuroscience*, *12*(12), 1859–1868. https://doi.org/10.1093/scan/nsx112

Supplementary Table 2. Imaging data acquisition

Dataset 1	Dataset 2
Structural and functional images were acquired at the Nuffield Department of Clinical Neurosciences (FMRIB, Oxford, UK) on a 3T scanner (Trio, Siemens)	Structural and functional images were acquired at Nuffield Department of Clinical Neurosciences (FMRIB, Oxford, UK) on a 3-Tesla whole-body scanner (Siemens

using a 24-channel head coil. Task functional images were acquired with a gradient echo T2*-weighted echo-planar sequence (TR 2000 ms, TE 30 ms, flip angle 70, 64x64 matrix, field of view 19.2^2 mm, voxel size 3x3x3mm). A total of 36 axial slices (3 mm thick, no gap) were sampled for whole-brain coverage excluding the cerebellum. Imaging data were acquired in four separate 120-volume runs of 4 min 02 s each. A high-resolution T1-weighted anatomical scan of the whole brain was acquired (256 x 256 matrix, voxel size 1 x 1 x 1 mm, TR = 1900 ms, TE = 3.97 ms, flip angle = 8°).	Magnetom Prisma) and a standard 32 channel coil. Tasks functional volumes were acquired using an interleaved, gradient-echo echo-planar pulse sequence with the following parameters with a gradient echo T2*-weighted echo-planar sequence (TR 2040 ms, TE 30 ms, flip angle 80, 64 × 64 matrix, field of view 192 mm, voxel size 3x3x3mm, parallel imaging GRAPPA, bandwidth = 1628 Hz/Px, PE = 2, and interleaved slice ordering). A total of 36 axial slices (3 mm thick, no gap) were sampled for whole-brain coverage excluding the cerebellum. Data were acquired in five runs of 180 volumes each. Each run lasted approximately 5 min 10 sec. Whole-brain anatomical images were acquired using a T1-weighted high-resolution magnetization prepared gradient echo (MPRAGE) sequence: TR = 1900 ms, TE = 3.97 ms, flip angle = 8°; field of view (FOV) = 192 mm, voxel size 1 × 1 × 1 mm.

Supplementary Table 3. Main behavioural results

In both datasets, participants were accurate in responding to stimuli (percent of correct responses varied from 82 to 96.9). A one-way ANOVA was employed to test the effect of interest on accuracy or response time performance. When appropriate, we performed Post Hoc tests (paired sample *t*-tests). We report the adjusted p-value for multiple comparisons using Holm Method (Holm, 1979). This method, in a stepwise way, computes the significance levels depending on the p-value based rank of hypotheses. MD- difference in means (the alternative hypothesis for these tests was that a true difference in means is not equal to 0).	
Accuracy performance	
Four separate ANOVAs were carried out on accuracy data for matched and mismatched trials to test a main effect of emotional relevance and a main effect of valence on accuracy performance. There was no main effect of emotional relevance (F(1,20)=0.00) or valence (F(2,40)=0.015, p=0.98, $\eta^2 = 0.00$) in matched or mismatched trials (F(1,20) =0.12; F(2,40)=0.66).	Two separate ANOVAs were performed to test the effects of reward value on accuracy performance in matched and mismatched trials. The results indicated no effects of reward value on accuracy in matched (F(1,18) = 0.98) and mismatched (F(1,18) =1.02) trials.
**Response time performance**	
In matched trials, there was a main effect of emotional relevance (F(1,20=62.64, p<.001, MD=-65.76, 95%CI [-87.45, -49.18]) and a main effect of valence (F(2,40)=29.70, p<0.001; *t*(20)= -6.83, p<0.001, MD = -69.38, 95%CI [-84.93, -47.35]); *t*(20)= -6.51, p<0.001, MD= -66.14, 95% CI [-82.97; -47.78]). Reaction times for happy and sad associations	In matched trials, there was a main effect of reward value (F(1,18) = 6.61, p=.019, MD = -46.34, 95% CI [-58.98, -37.12] indicating that participants were faster in responding to shapes associated with high reward value compared to shapes associated with low reward value.<br><br>In mismatched trials, the difference between shapes associated with high and low reward

were faster compared to associations with neutral emotional expression (*t*(20)= -6.51, p<0.001, MD= -66.14, 95% CI [-82.97; -47.78]). The difference between happy and sad associations were not significant (*t*(20)= -0.32, p=0.75).<br><br>In mismatched trials, there was no main effect of emotion relevance (F(1,20)=0.07). A main effect of valence was significant (F(2,40)=13.64, p<.001). A Post Hoc analysis indicated that participants' responses were faster for shapes paired with happy emotional expression compared to shapes paired with sad (t(20)=-5.18, MD = -25.22, p_{holm} <.001) and neutral (t(20)=-3.39, MD=-22.85, p_{holm} = .006)	values was non-significant (F(1,18) =1.12, p=.69, MD = 2.98, 95% CI [-12.65, 6.11].

Supplementary Figure 1. Examples of quality control (QC) of functional connectivity (FC). A-dataset 1, B-dataset 2, C-example of QC of individual dataset

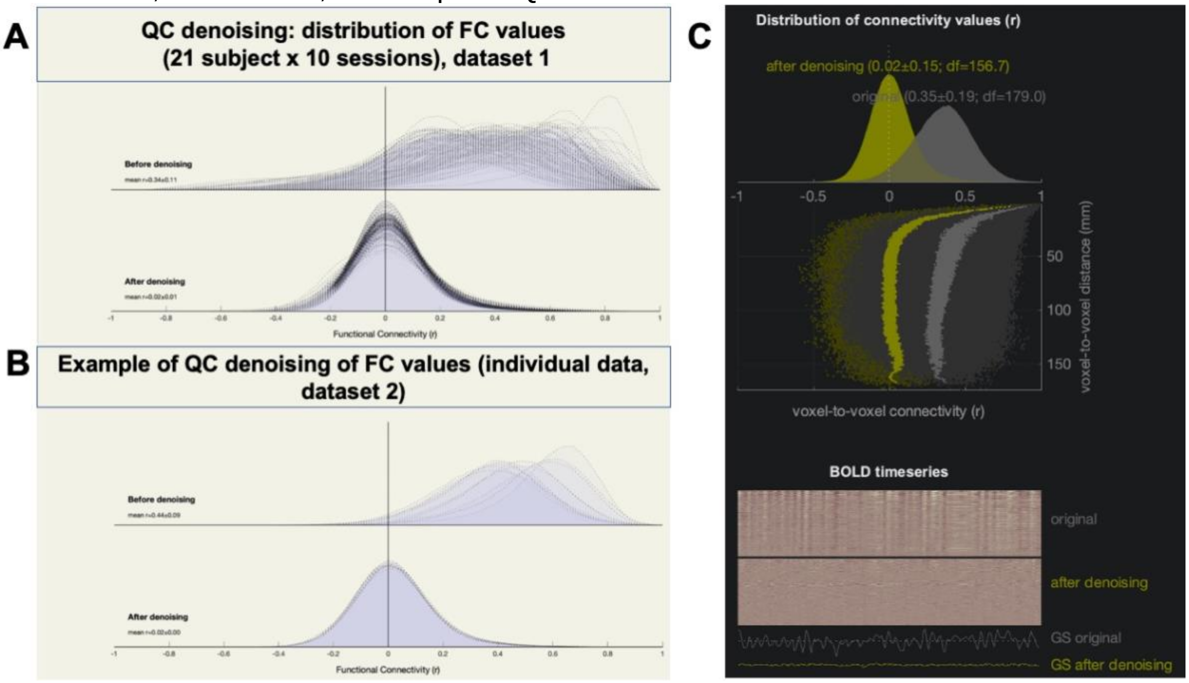

Supplementary Figure 2. Set of large-scale networks

DefaultMode.MPFC (1,55,-3)
DefaultMode.LP (L) (-39,-77,33)
DefaultMode.LP (R) (47,-67,29)
DefaultMode.PCC (1,-61,38)
SensoriMotor.Lateral (L) (-55,-12,29)
SensoriMotor.Lateral (R) (56,-10,29)
SensoriMotor.Superior (0,-31,67)
Visual.Medial (2,-79,12)
Visual.Occipital (0,-93,-4)
Visual.Lateral (L) (-37,-79,10)
Visual.Lateral (R) (38,-72,13)
Salience.ACC (0,22,35)
Salience.AInsula (L) (-44,13,1)
Salience.AInsula (R) (47,14,0)
Salience.RPFC (L) (-32,45,27)
Salience.RPFC (R) (32,46,27)
Salience.SMG (L) (-60,-39,31)
Salience.SMG (R) (62,-35,32)
DorsalAttention.FEF (L) (-27,-9,64)
DorsalAttention.FEF (R) (30,-6,64)
DorsalAttention.IPS (L) (-39,-43,52)
DorsalAttention.IPS (R) (39,-42,54)
FrontoParietal.LPFC (L) (-43,33,28)
FrontoParietal.PPC (L) (-46,-58,49)
FrontoParietal.LPFC (R) (41,38,30)
FrontoParietal.PPC (R) (52,-52,45)
Language.IFG (L) (-51,26,2)
Language.IFG (R) (54,28,1)
Language.pSTG (L) (-57,-47,15)
Language.pSTG (R) (59,-42,13)
Cerebellar.Anterior (0,-63,-30)
Cerebellar.Posterior (0,-79,-32)

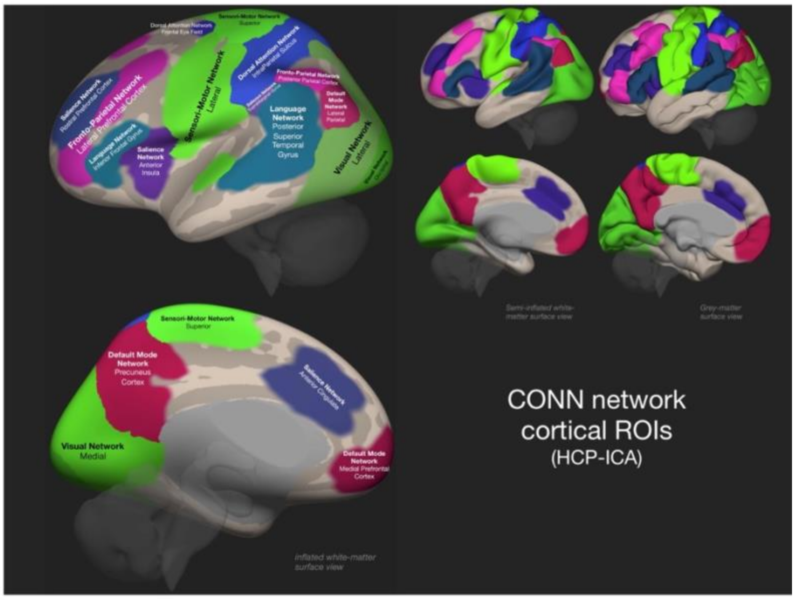

Supplementary Table 4. The results of Network Based Statistic analysis with varied connectivity (‘height’) thresholds for contrast [***high reward > low-reward***] (p-value for each component was FWE corrected). NS denotes that a component did not survive the FWE correction.

Height threshold (p<)	Connections comprising a component	Component statistics		
		mass	size	p-FWE value
.05 - .009	NS			
.009 - .002	NS			
.001 - .0009	DMN.MPFC->FPN.PPC(R)<br>DMN.MPFC->SN.ACC<br>DMN.MPFC->FPN(L)<br>FPN.PPC(L)->DAN.IPS(L)<br>SN.ACC->DAN.IPS(L)	187.61	5	0.001-0.01
.0001 - .00009	DMN.MPFC->FP.PPC(R)<br>DMN.MPFC->SN.ACC<br>SN.ACC->DAN.IPS(L)	147.00	3	.0008 -.004
.00008 -	DMN.MPFC->FPN.PPC(R)	68.71	1	.004

Supplementary Table 5. The results of Network Based Statistic analysis with varied connectivity (‘height’) thresholds for contrast [***emotion (happy+sad) > neutral***] (p-value for each component was FWE corrected). NS denotes that a component did not survive the FWE correction.

Height threshold (p<)	Connections comprising a component	Component statistics		
		mass	size	p-FWE value
.05 - .009	NS			
.001 - .0007	DMN.MPFC->DAN.FEF(L)<br>DMN.MPFC->DAN.FEF(R)<br>DMN.MPFC->SN.RPFC(L)<br>SN.RPFC(L) ->Language.IFG(L)<br>Language.IFG(L)-> SN.RPFC(L)<br>DMN.MPFC->FPN.PPC(L)	116.67	6	.006-.004

.0006 - .0002	DMN.MPFC->DAN.FEF(L)<br>DMN.MPFC->DAN.FEF(R)<br>DMN.MPFC->SN.RPFC(L)	66.09	3	.01-.02
.0001	NS			

Supplementary Table 6. The results of Network Based Statistic analysis with varied 'height' thresholds for contrast [***sad > neutral***] (p-value for each component was FWE corrected). NS denotes that a component did not survive the FWE correction.

Height threshold (p<)	Connections comprising a component	Component statistics		
		mass	size	p-FWE value
.05 - .01	NS			
.009 - .005	NS			
.004 - .0006	DMN.MPFC->DAN.FEF(L)<br>DMN.MPFC->DAN.FEF(R)<br>DMN.MPFC->Visual.Medial<br>Visual.Media-> DMN.MPFC	82.91	4	.006
.0005 - .0001	DMN.MPFC->DAN.FEF(L)<br>DMN.MPFC->DAN.FEF(R)	54.13	2	.022
.00009 - .00003	DMN.MPFC->DAN.FEF(L)<br>DMN.MPFC->DAN.FEF(R)	54.13	2	.007
.00002	DMN.MPFC->DAN.FEF(L)	48.32	1	.007
.00001	NS			

Supplementary Table 7. The results of Network Based Statistic analysis with varied 'height' thresholds for contrast [***happy > neutral***] (p-value for each component was FWE corrected). NS denotes that a component did not survive the FWE correction.

Height threshold (p<)	Connections comprising a component	Component statistics		
		mass	size	p-FWE value
.05 - .01	NS			
.009 - .004	NS			
.003 - .002	DMN.MPFC->SN.RPFC(L)<br>DMN.MPFC-> FP.PPC(L)<br>DMN.MPFC->Language.pSTG(R)	78.93	2	.032
.001 - .0001	DMN.MPFC->SN.RPFC(L)<br>DMN.MPFC-> FPN.PPC(L)	56.50	2	.034
.00009 - .00003	DMN.MPFC->SN.RPFC(L)<br>DMN.MPFC-> FPN.PPC(L)	58.04	2	.034
.00002	DMN.MPFC-> FPNPPC(L)	29.90	1	.008
.00001	NS			
